# Neural Correlates of Subjective Awareness for Natural Scene Categorization of Color Photographs and Line-Drawings

**DOI:** 10.3389/fpsyg.2017.00210

**Published:** 2017-02-15

**Authors:** Qiufang Fu, Yong-Jin Liu, Zoltan Dienes, Jianhui Wu, Wenfeng Chen, Xiaolan Fu

**Affiliations:** ^1^State Key Laboratory of Brain and Cognitive Science, Institute of Psychology, Chinese Academy of SciencesBeijing, China; ^2^University of Chinese Academy of SciencesBeijing, China; ^3^Tsinghua National Laboratory for Information Science and Technology, Department of Computer Science and Technology, Tsinghua UniversityBeijing, China; ^4^Sackler Centre for Consciousness Science, School of Psychology, University of SussexBrighton, UK; ^5^College of Psychology and Sociology, Shenzhen UniversityShenzhen, China

**Keywords:** visual awareness, subjective measures, objective measures, natural scene categorization, Perceptual Awareness Scale (PAS)

## Abstract

It remains controversial whether visual awareness is correlated with early activation indicated by VAN (visual awareness negativity), as the recurrent process hypothesis theory proposes, or with later activation indicated by P3 or LP (late positive), as suggested by global workspace theories. To address this issue, a backward masking task was adopted, in which participants were first asked to categorize natural scenes of color photographs and line-drawings and then to rate the clarity of their visual experience on a Perceptual Awareness Scale (PAS). The interstimulus interval between the scene and the mask was manipulated. The behavioral results showed that categorization accuracy increased with PAS ratings for both color photographs and line-drawings, with no difference in accuracy between the two types of images for each rating, indicating that the experience rating reflected visibility. Importantly, the event-related potential (ERP) results revealed that for correct trials, the early posterior N1 and anterior P2 components changed with the PAS ratings for color photographs, but did not vary with the PAS ratings for line-drawings, indicating that the N1 and P2 do not always correlate with subjective visual awareness. Moreover, for both types of images, the anterior N2 and posterior VAN changed with the PAS ratings in a linear way, while the LP changed with the PAS ratings in a non-linear way, suggesting that these components relate to different types of subjective awareness. The results reconcile the apparently contradictory predictions of different theories and help to resolve the current debate on neural correlates of visual awareness.

## Introduction

Recently, an increasing amount of attention has been given to neural correlates of visual awareness, i.e., the subjective experience of seeing. Neural imaging studies have revealed that a distributed system, including the primary visual cortex (V1), V2, temporal, parietal, and frontal areas, plays a crucial role in the generation of visual awareness (see Koch et al., 2016 for a review; Rees, 2001; Vuilleumier et al., 2002; Salminen-Vaparanta et al., 2012). Electrophysiological studies have shown that an early positive enhancement (P1) over occipital regions that were approximately 100 ms from the stimulus onset, a negative difference wave (visual awareness negativity, VAN) at the posterior regions typically occurring 150–250 ms from the stimulus onset, and a P3 or a late positive (LP) wave occurring 300 ms from the stimulus onset were probably correlated with the emergence of visual awareness (see Koivisto and Revonsuo, 2010; Revonsuo and Koivisto, 2010; Naccache et al., 2016; contrast Silverstein et al., 2015). However, there is no agreement on what the identity of the neural indicator is for the emergence of visual awareness. For example, the N1 component (e.g., Jiang et al., 2013), the P2 component (e.g., Melloni et al., 2011), and the N2 component (e.g., Koivisto and Revonsuo, 2003) have been also argued to be related to visual awareness. The identity of the neural indicator for the emergence of visual awareness is still unclear.

One of the challenges of addressing this issue is the measurement of the status of visual awareness. There are two measures of awareness or consciousness in the literature: objective and subjective measures. With objective measures, the ability to discriminate *features of the world* is taken to indicate whether knowledge is conscious (Fu et al., 2008; Koivisto et al., 2008). An inability to discriminate reflects the absence of conscious knowledge. Using objective measures, P1 and VAN might be found to be related to visual awareness. For example, in a two-alternative matching to sample task, participants were asked to determine whether a briefly presented “sample word” was the same or different from a second “test word” by pressing one of two keys. Melloni et al. (2007) found that a P1-like component was significantly different between visible and invisible words, indicating that the P1 component was the earliest event-related potential (ERP) component that correlated with visual awareness. Pins and Ffytche (2003) also found a P1 effect for visual awareness, but this P1 correlate was not been observed in other studies (e.g., Sergent et al., 2005; Koivisto et al., 2008; Melloni et al., 2011). Moreover, Koivisto et al. (2008) compared the neural activities for hit trials (aware) with those for miss trials (unaware) and found that the posterior VAN was an earlier ERP correlate of visual awareness. The posterior VAN correlate has been observed in other studies (e.g., Koivisto and Revonsuo, 2003; Genetti et al., 2009).

With subjective measures, the ability to report or discriminate *mental states* is taken to indicate whether the knowledge is conscious (Fu et al., 2008; Lamy et al., 2009). If a person says he does not see or he has no experience of the stimulus, the knowledge revealed in the behavioral forced-choice response is unconscious by these measures. Using subjective measures, P3 or LP might be found to be related to visual awareness. For example, Lamy et al. (2009) compared the neural activities for correct trials in which participants reported they had seen the stimulus (aware-correct) and correct trials in which participants reported that they merely guessed (unaware-correct). They found that only an enhanced P3 effect with a widely distributed topography was related to subjective awareness. Similarly, Del Cul et al. (2007) also found that only the P3 amplitude distinguished visible and invisible stimuli in subjective reports. Moreover, although the studies using objective measures found VAN to be related to visual awareness, most of them also found P3 or LP (later positive) effects in visual awareness (e.g., Koivisto et al., 2008; Genetti et al., 2009).

Obviously, different measures lead to different findings in the neural correlates of visual awareness, but more importantly, they are based on different theories of consciousness. For example, subjective measures are consistent with the assumption of the higher-order thought (HOT) theory that states that a distinguishable characteristic of a conscious state is that an individual can report the state (Rosenthal, 2005; Lau and Rosenthal, 2011). According to the HOT theory, the conscious status of a mental state is most naturally measured by subjective measures. Meanwhile, the global workspace (GWS) theory takes the dynamic long-distance synchronization as a key feature for conscious condition (Dehaene and Naccache, 2001). According to the GWS theory, objective measures could be satisfied by local processors without information entering the workspace. Information in the workspace would be available to higher order thoughts, so the GWS theory naturally motivates subjective measures. In this respect, the HOT and GWS theory are similar. In contrast, a recent recurrent processing hypothesis states that localized recurrent processing is sufficient for consciousness, and while not entirely obvious theoretically, this approach has been used to motivate objective measures (Lamme, 2010). But most generally, the local recurrent processing approach attempts to move away from behavioral or introspective methods in the measure of consciousness.

The widely distributed P3 or LP correlate of visual awareness is in line with the prediction of the GWS theory, which assumes that visual awareness correlates with later activation in the fronto-parietal cortices; the more local posterior VAN correlate of visual awareness is in line with the recurrent processing hypothesis, which proposes that visual awareness correlates with early activation in the visual cortex (Windey et al., 2013; Andersen et al., 2015; Koivisto and Grassini, 2016). In addition, the GWS theory assumes that visual awareness is dichotomous, while the recurrent processing hypothesis assumes that visual awareness is graded (Windey et al., 2013; Andersen et al., 2015; Koivisto and Grassini, 2016). It remains controversial whether visual awareness is correlated with early activation indicated by VAN, as the recurrent process hypothesis theory proposes, or with later activation indicated by P3 or LP, as suggested by the GWS and HOT theories, and whether the neural indicator is correlated with visual awareness in a dichotomous or graded way.

Interestingly, Timmermans et al. (2010) argued that studies supporting the GWS theory generally use complex stimuli (e.g., Del Cul et al., 2007, 2009), whereas studies supporting the recurrent processing hypothesis mostly use simple stimuli (e.g., Fahrenfort et al., 2007). Moreover, Windey et al. (2013) found that simple task produced evidence for graded visual awareness, whereas more complex task produced more dichotomous visual awareness. Thus, the findings about the neural correlates of visual awareness could be influenced by stimulus or task complexity. To the best of our knowledge, no study has adopted complex stimuli such as natural scenes to explore neural correlates of visual awareness. Natural scenes are rather complex, but people can categorize them quickly and accurately, even with little or no attention (Li et al., 2002; Rousselet et al., 2002; Feifei et al., 2005; Otsuka and Kawaguchi, 2007), and it has been claimed that the integration of scene elements can be achieved without awareness (Mudrik et al., 2011; Mudrik and Koch, 2013), although there is inconsistent evidence for this (Faivre and Koch, 2014; Moors et al., 2016). Moreover, recently, a fMRI study found that natural scene categorization of line-drawings generated similar neural activation as color photographs, although color photographs includes more features such as color and contexts than line-drawings (Walther et al., 2011).

Therefore, to further examine what are the neural correlates of visual awareness, we adopted natural scenes of both color photographs and line-drawings in a backward masking task. In the backward masking task, the stimulus duration was identical, i.e., the physical stimulus was identical, and the interstimulus interval (ISI) between the stimulus and the mask was manipulated. It has been demonstrated that masking has little effect on the feedforward processing of a stimulus from low to high visual areas, but interferes with the recurrent processing of the stimulus from high to low visual areas that is crucial for the emergence of visual awareness (Rolls et al., 1999; Lamme et al., 2002; Lamme, 2010). To measure the status of visual awareness, we used subjective measures because they are motivated by two major psychological approaches to consciousness, that is, higher order and GWS approaches. Specifically, we used the Perceptual Awareness Scale (PAS), in which participants were asked to rate the clarity of their visual experience using a 4-point scale with the elements: no experience, brief glimpse, almost clear image, and clear image (Ramsøy and Overgaard, 2004; Sandberg et al., 2010; see also Dienes and Seth, 2010). The interpretation of PAS depends on its theoretical relation to awareness of the relevant stimulus characteristics. On one view, each PAS rating represents an increase in visual awareness in a graded way (e.g., Ramsøy and Overgaard, 2004). On another view, the distinction between, on the one hand, brief glimpse, in which the subject claims not to have seen anything of relevance, and almost clear image, on the other hand, marks a distinct change in the conscious awareness of information relevant to the task discrimination (Dienes and Seth, 2010). On the first view, a correlate of awareness should vary linearly with the PAS ratings; on the second view, relevant components should vary non-linearly with the PAS ratings. Further, if subjective visual awareness correlates with early activation in the visual cortex, the posterior VAN should vary with the PAS ratings in an appropriate way; or else, if long-distance activation among brain areas is additionally required for the emergence of subjective awareness, a widely distributed P3 or LP would vary with the PAS ratings in an appropriate way.

## Materials and Methods

### Participants

Twenty-two college students (11 males and 11 females; mean age = 21.82 years, *SD* = 2.34 years) participated in this study. All of them had normal or corrected-to-normal vision, and none of them had any history of neurological or psychiatric diseases. This experiment was approved by the committee for the protection of subjects at the Institute of Psychology, Chinese Academy of Sciences. All students gave written informed consent and were paid for their attendance.

### Stimuli and Apparatus

**FIGURE 1:**
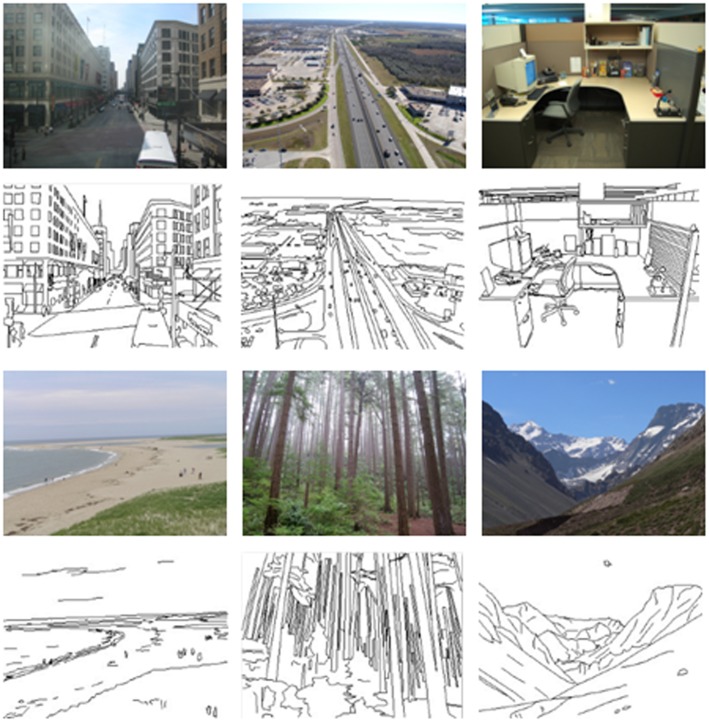
**Examples of six natural scene categories of color photographs and line-drawings**.

The stimuli were images of six natural scene categories: beaches, city streets, forests, highways, mountains, and offices (see **Figure 1**). Each image had a resolution of 320 pixels × 240 pixels in two versions: color photographs and line-drawings. The line-drawings were produced by trained artists by tracing contours in the color photographs (see Walther et al., 2011). Each category had 76 to 80 different images, for a total of 475. The masks were two white noise images at two different spatial scales: one was generated at the resolutions of 320/240, and the other was generated at the resolutions of 20/15 and then resized to 320/240 pixels. Each mask was also resized to 320 pixels × 240 pixels in two versions: one was in color and the other was in grayscale. The stimulus was presented against a silver gray background on a CRT monitor with a resolution of 1280 pixels × 768 pixels and a refresh rate of 75 Hz. The distance from the participants to the screen was approximately 60 cm, and the horizontal and vertical visual angles were approximately 8.7 and 8.2 degrees, respectively.

### Procedure

Each trial began with a black fixation cross at the center of the screen for 500–950 ms at random. Then, an image was presented for 13 ms, and followed by two masks for 100 ms. Each mask was shown for 50 ms and the sequence of the two masks was randomly assigned. For color photographs, the masks were in color, and for line-drawings, the masks were in grayscale. The ISI between the image and the mask was 0, 13, 26, or 200 ms at random. After the masks, a blank was presented for 500 ms and then six category names appeared on the screen from left to right. Participants were asked to report the category of the presented image by pressing a corresponding key on the keyboard. The keys D, F, G, H, J, and K corresponded to the locations of the six category names. To prevent participants from preparing for their response before the appearance of the category names, the locations of the six category names were randomly assigned for each trial. There was no feedback about the response. After the response, they were asked to rate their experience of the stimulus, i.e., how clearly did they see the image, with four possible options from left to right on the PAS scale [(1) no experience; (2) brief glimpse; (3) almost clear experience; (4) clear experience] by pressing a corresponding key. Participants were told to select the option according to their true feelings and then to press the space key to start the next trial when they were ready. There were approximately 95 trials in each block, in which type of image (color photographs vs. line-drawings), natural scene category, and ISI were counterbalanced. There were at least 30 s of rest between any two blocks. The experiment included 10 blocks, for a total of 950 trials.

### EEG Recording and Analysis

Electroencephalograph (EEG) was recorded with a NeuroScan Synamps amplifier using a custom 64-channel cap. The left mastoid was used as an on-line reference and the algebraic average of left and right mastoids was used as off-line re-reference. Two pairs of electrodes placed 1 cm above and below one eye and 1 cm lateral from the outer canthi of both eyes were used to monitor blinks and other eye movements. The EEG signals were amplified by using a band pass of 0.05 to100 Hz, with a sampling rate of 500 Hz. Electrode impedances were kept below 5 kΩ. For the analysis of ERPs, the continuous EEG signals were low pass filtered, with a cutoff frequency at 30 Hz, and then segmented in series of epochs of 800 ms. Each epoch started 100 ms before the stimulus onset. Baseline correction was performed over the 100 ms window before the stimulus presentation. Trials containing voltages exceeding ± 80 μV were rejected. The average rejection rate was only 2.17%.

ERPs for correct trials were averaged separately for the different experience ratings of color photographs and line-drawings. Based on previous studies (for review, see Koivisto and Revonsuo, 2010; Melloni et al., 2011), the mean amplitudes of the ERPs were analyzed for P1 (100–130 ms), N1 (140–200 ms), P2 (150–190 ms), N2 (210–310 ms), VAN (240–300 ms), P3a (370–470 ms), and LP or P3 (420–570 ms). The time windows for the statistical analysis of the ERPs were determined on the basis of the grand average waves. The mean amplitudes were computed over groups of electrodes representative of the topography of each component. For P1, N1, and VAN, a group of occipital electrodes (CB1, O1, Oz, O2, and CB2) was selected; for P2, N2, and P3a, a group of fronto-central electrodes (F3, Fz, F4, FC3, FCz, FC4, C3, Cz, and C4) was selected; for LP, a group of occipito-parietal electrodes (P3, Pz, P4, PO3, POz, PO4, O1, Oz, and O2) was selected. Each was subjected to repeated two-way ANOVA with the type of image (color photographs vs. line-drawings) and experience ratings (no experience vs. brief glimpse vs. almost clear experience vs. clear experience) as within-subject variables.

## Results

### Inferential Strategy

For all tests *p*-values are reported; in addition, for all one degree of freedom tests, Bayes factors, *B*, are reported. Bayes factors (*B*) were used to assess strength of evidence for H1 relative to H0 (Wagenmakers et al., 2015). Unlike null-hypothesis significance testing, Bayes factors have the advantage of distinguishing sensitive evidence for H0 from not much evidence at all (relative to the range of effects that could plausibly be obtained on H1). A *B* of above 3 indicates evidence for the alternative hypothesis and below 1/3 evidence for the null hypothesis. *B*s between 3 and 1/3 indicate data insensitivity in distinguishing H0 and H1 (see Dienes, 2014; cf Jeffreys, 1939). Thus we will report that there was no effect only when *B* < 1/3, that is, when there is evidence for H0 over H1. Here, *B*_N(0,_*_x_*_)_ refers to a Bayes factor in which the predictions of H1 were modeled as normal distribution with an SD of *x* (see Dienes, 2014), where *x* scales the size of effect that could be expected. If a rough maximum effect can be determined, *x* can be set as half that value (Dienes, 2014).

For proportions of different PAS ratings, with four ratings the average proportion was 0.25, so we report *B*_N(0,0.25)_. For accuracy of classification, as the range from baseline (i.e., chance level 0.17) to 1 was 0.83, a difference of 0.83 is the maximum difference that could be expected between any two conditions. Thus, we took half of 0.83 as the SD and report *B*_N(0,0.42)_. For ERP results, we took half of the roughly average difference between maximal and minimal amplitude of the ERP component over color photographs and line-drawings as *x* for each component. Note that the difference between maximal and minimal amplitude was never itself tested, so there is no double counting (Jeffreys, 1939). As positive trend coefficients summed to 1 and negative to -1, the expected size of linear trends is the same as a simple difference. Thus, for trend contrasts, H1 was modeled as a normal with an SD equal to half of the roughly average difference between maximal and minimal amplitudes of the ERP component, as just mentioned. With these models of H1, it so happened that whenever *p* < 0.05, then *B* > 3, and vice versa. There is no guarantee of this correspondence (Lindley, 1957), but in the current case significance testing and Bayesian analyses agreed when indicating that there was an effect.

### Behavioral Results

**FIGURE 2:**
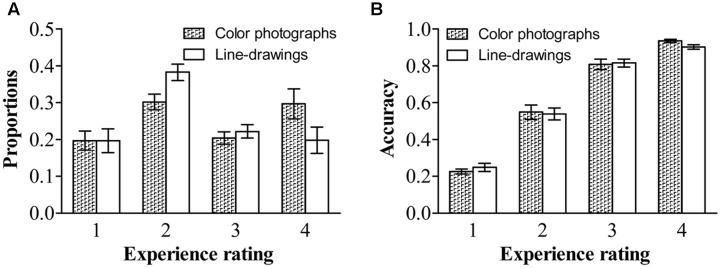
**Proportions and accuracies of each experience rating for each type of image. (A)** Proportions of each experience rating for each type of image. (B) Accuracies of each experience rating for each type of image. Error bars depict standard errors. Note that 1 refers to “no experience,” 2 refers to “brief glimpse,” 3 refers to “almost clear experience,” and 4 refers to “clear experience” on the Perceptual Awareness Scale (PAS).

As in previous studies (e.g., Ramsøy and Overgaard, 2004; Del Cul et al., 2007; Railo et al., 2015), we treated the experience rating as a within-subject factor. **Figure 2** shows the proportion and accuracy for each experience rating for both types of images. Repeated ANOVA on proportions, with experience ratings and type of image as within-subject variables, revealed a significant effect of experience ratings, *F*(1.62,33.97) = 4.34, *p* < 0.01, $\eta_{p}^{2}$ = 0.17, and a significant experience ratings by type of image interaction, *F(*3,63) = 22.55, *p* < 0.001, $\eta_{p}^{2}$ = 0.52. People reported having a “brief glimpse” less often for color photographs than for line-drawings, *t*(21) = -7.37*, p* < 0.001, *dz* = 1.61, *B*_N(0,0.25)_ = 7.13 × 10^10^; but reported having a “clear experience” more often for color photographs than for line-drawings, *t*(21) = 6.69*, p* < 0.001, *dz* = 1.46, *B*_N(0,0.25)_ = 9.07 × 10^8^. There were no differences between color photographs and line-drawings in the proportion of time they reported having “no experience,” *t*(21) = -0.03, *p* = 0.98, *B*_N(0,0.25)_ = 0.08, nor in the proportion of “almost clear experience” reports, *t*(21) = -1.43*, p* = 0.17, *B*_N(0,0.25)_ = 0.32. Overall, the above results provided substantial evidence that people had subjective experiences with more felt clarity of color photographs than of line-drawings.

Repeated ANOVA on accuracy, with experience ratings and type of image as within-subject variables, revealed only a significant effect of experience ratings, *F*(1.83,38.86) = 299.49, *p* < 0.001, $\eta_{p}^{2}$ = 0.93. For both types of images, the accuracy was higher for a “clear image” than for an “almost clear image,” *t*(21) = 4.81, *p* < 0.001, *dz* = 1.05, *B*_N(0,0.42)_ = 3.66 × 10^20^, higher for an “almost clear image” than for a “brief glimpse,” *t*(21) = 13.05, *p* < 0.001, *dz* = 2.85, *B*_N(0,0.42)_ = 1.58 × 10^35^, and higher for a “brief glimpse” than for “no experience,” *t*(21) = 9.90, *p* < 0.001, *dz* = 2.16, *B*_N(0,0.42)_ = 1.47 × 10^4^. There was no effect of type of image, *F*(1,21) = 0.09, *p* = 0.77, $\eta_{p}^{2}$ = 0.004, *B*_N(0,0.42)_ = 0.04. The two-way interaction did not reach significance, *F*(3,63) = 2.17, *p* = 0.10, $\eta_{p}^{2}$ = 0.09. For both color photographs and line-drawings, the accuracy was above chance (0.17) for “no experience,” *t*(21) = 4.11, *p* < 0.001, *dz* = 0.88, *B*_N(0,0.42)_ = 2.85 × 10^58^, *t*(21) = 3.56, *p* < 0.01, *dz* = 0.76, *B*_N(0,0.42)_ = 3.51 × 10^26^. That is, people could correctly classify natural scenes even when they reported that they did not see them at all.

### ERP Results

**FIGURE 3:**
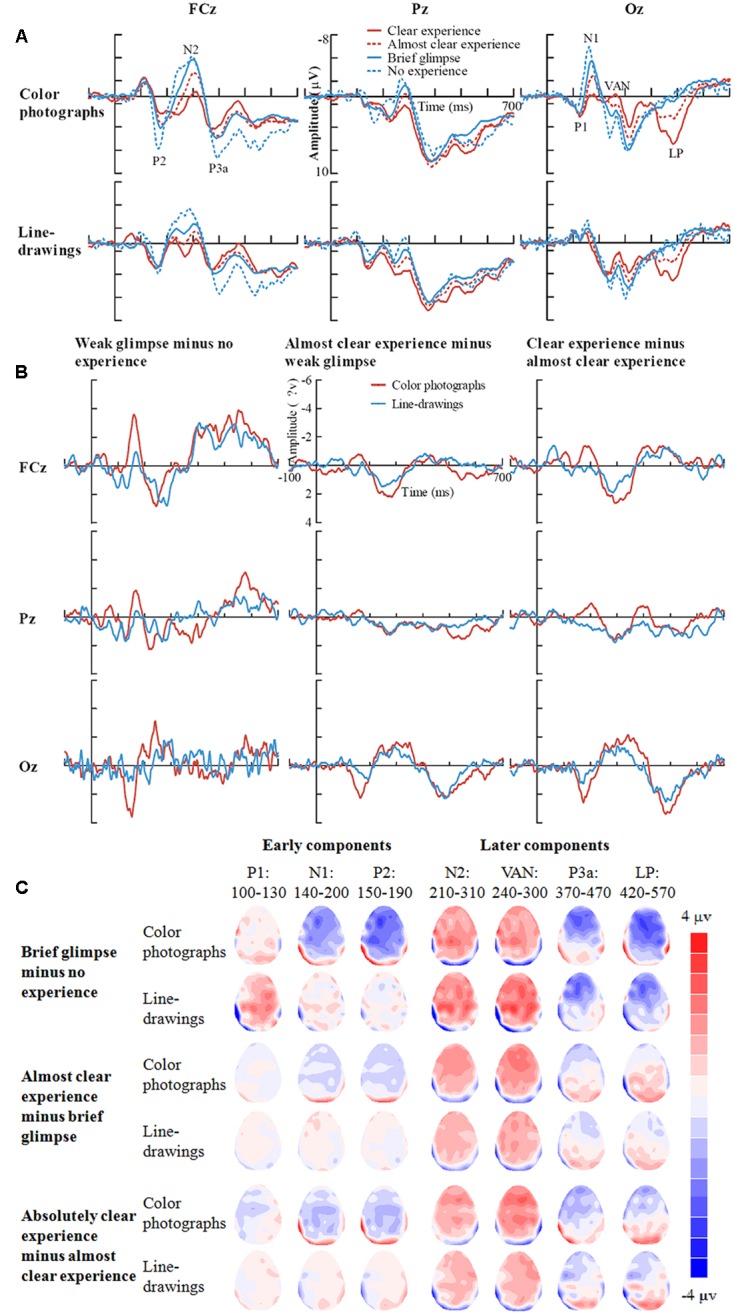
**Grand-average ERPs for each experience rating for color photographs and line-drawings**. **(A)** Grand-average ERPs of correct trials at electrodes FCz, Pz, and Oz for each experience rating for color photographs and line-drawings. **(B)** ERP differences between every two adjacent experience ratings for color photographs and line-drawings. **(C)** The scalp topography of the P1, N1, P2, N2, VAN, P3a, and LP, trials of the relatively higher experience ratings minus trials of the adjacent lower experience ratings separately for color photographs and line-drawings.

**FIGURE 4:**
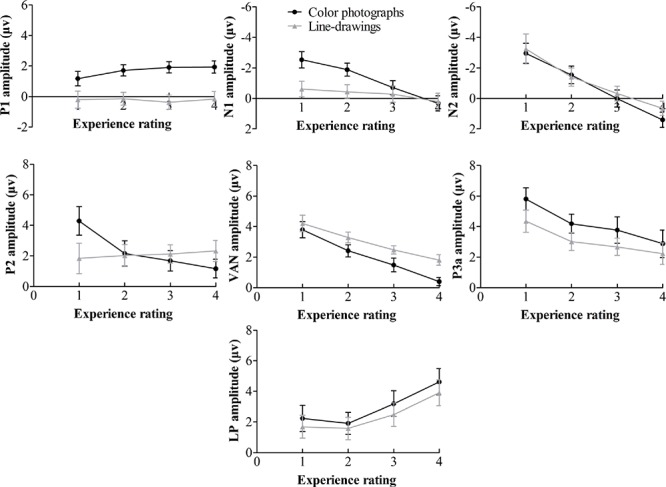
**Mean amplitudes of each component on experience rating for color photographs and line-drawings**. Error bars depict standard errors. Note that 1 refers to “no experience,” 2 refers to “brief glimpse,” 3 refers to “almost clear experience,” and 4 refers to “clear experience” on the PAS.

Inspection of ERPs revealed seven major components: P1, N1, P2, N2, VAN, P3a, and LP (or P3). **Figure 3** shows the ERP data for each condition. **Figure 4** shows the mean amplitudes of each component for each condition. To evaluate which components were correlated with subjective awareness, repeated two-way ANOVA with experience ratings (no experience vs. brief glimpse vs. almost clear experience vs. clear experience) and type of images (color photographs vs. line-drawings) as within-subject variables was conducted for the mean amplitude of each component. Moreover, as “brief glimpse” involves some visual experience, but not of anything relevant to making the task discrimination, the difference between “brief glimpse” and “almost clear image” is when perceptual contents go from largely unconscious and to largely conscious. Even on a graded consciousness view (Ramsøy and Overgaard, 2004), this particular contrast represents a distinction in amount of conscious awareness. Thus, to further examine whether each component was a real marker of the conscious status of knowledge of a stimulus, the planned comparison between “brief glimpse” and “almost clear image” were conducted for the mean amplitude of each component.

**Table 1** summarizes significant results of the ANOVAs. As observed from **Table 1**, for posterior P1 amplitudes, the effect of the experience ratings and interaction did not reach significance (*p*s > 0.31). The planned comparison revealed that there was evidence for no difference between “brief glimpse” and “almost clear experience” for color photographs, *t*(21) = -1.24, *p* = 0.23, *dz* = 0.26, *B*_N(0,3)_ = 0.26, and marginal evidence that there was no difference for line-drawings, *t*(21) = 1.46, *p* = 0.16, *dz* = 0.31, *B*_N(0,3)_ = 0.35. That is, the posterior P1 amplitude did not always relate to subjective awareness.

**Table 1:** Significant results of the two-way repeated ANOVAs performed over the amplitude of each component, considering experience ratings and type of images.

	Experience ratings		Type of image		Experience ratings by type of image	
	*F*	$\eta_{p}^{2}$	*F*	$\eta_{p}^{2}$	*F*	$\eta_{p}^{2}$
Posterior P1			38.49^∗∗∗^	0.65		
Posterior N1	25.65^∗∗∗^	0.55	5.54^∗^	0.21	7.74^∗∗∗^	0.27
Anterior P2	5.21^∗∗^	0.20			6.62^∗∗^	0.24
N2	27.04^∗∗∗^	0.56				
VAN	27.74^∗∗∗^	0.57	25.14^∗∗∗^	0.55		
P3a	11.13^∗∗^	0.35	6.20^∗∗^	0.23		
LP	14.68^∗∗∗^	0.41	5.93^∗∗^	0.22		

*In each ANOVA, we report F-values with significance and η_*p*_^*2*^. ^∗^*p* < 0.05, ^∗∗^*p* < 0.01, ^∗∗∗^*p* < 0.001*.

For posterior N1 and anterior P2 amplitudes, the two-way ANOVAs revealed significant experience rating effects which were modulated by the type of image. Further analysis revealed that for color photographs only, the amplitudes of posterior N1 and anterior P2 significantly varied with visual awareness, *F*(1.86,39.08) = 23.02, *p* < 0.001, $\eta_{p}^{2}$ = 0.52, *F*(2.09,43.81) = 10.54, *p* < 0.001, $\eta_{p}^{2}$ = 0.33, but did not for line-drawings (*p*s > 0.12). For N1, the planned comparison revealed that “brief glimpse” differed from “almost clear experience” for color photographs, *t*(21) = -5.85, *p* < 0.001, *dz* = 1.25, *B*_N(0,3)_ = 4.46 × 10^6^, but not for line-drawings, *t*(21) = -0.81, *p* = 0.43, *dz* = 0.17, *B*_N(0,3)_ = 0.18. For P2, the planned comparison revealed that there was no evidence for whether or not “brief glimpse” differed from “almost clear experience” for color photographs, *t*(21) = 1.14, *p* = 0.27, *dz* = 0.24, *B*_N(0,4)_ = 0.47, but there was substantial evidence for no difference for line-drawings, *t*(21) = -0.07, *p* = 0.94, *dz* = 0.01, *B*_N(0,4)_ = 0.08. Thus, the results indicated that the posterior N1 and anterior P2 amplitudes did not always correlate with subjective awareness.

For the later components N2, VAN, P3a, and LP, the two-way ANOVAs revealed significant experience rating effects (all *p*s < 0.01), which were not significantly influenced by the type of images (all *p*s > 0.19). For N2, the planned comparison revealed that “brief glimpse” differed from “almost clear experience” for both color photographs, *t*(21) = -4.03, *p* = 0.001, *dz* = 0.86, *B*_N(0,4)_ = 8.20 × 10^2^, and line-drawings, *t*(21) = -3.42, *p* < 0.01, *dz* = 0.73, *B*_N(0,4)_ = 69.39. For VAN, “brief glimpse” differed from “almost clear experience” for both color photographs, *t*(21) = 3.12, *p* < 0.01, *dz* = 0.67, *B*_N(0,4)_ = 23.89, and line-drawings, *t*(21) = 4.06, *p* < 0.001, *dz* = 0.87, *B*_N(0,4)_ = 2.35 × 10^3^. However, for P3a, there was not much evidence for the difference between “brief glimpse” and “almost clear experience” either way for either color photographs, *t*(21) = 0.84, *p* = 0.41, *dz* = 0.18, *B*_N(0,4)_ = 0.35, or line-drawings, *t*(21) = 1.08, *p* = 0.29, *dz* = 0.23, *B*_N(0,4)_ = 2.59. Finally, for LP, there was a difference between “brief glimpse” and “almost clear experience” for both color photographs, *t*(21) = 3.95, *p* = 0.001, *dz* = 0.84, *B*_N(0,3)_ = 6.60 × 10^2^, and line-drawings, *t*(21) = 2.11, *p* < 0.05, *dz* = 0.45, *B*_N(0,3)_ = 3.24. That is, the results suggested that the N2, VAN, and LP amplitudes could be related to the emergence of subjective awareness, while the evidence for P3a amplitude marking a change in subjective awareness was insensitive.

To further examine how the later components correlated with the subjective awareness, we used “no experience,” “brief glimpse,” and “almost clear experience” to calculate the linear trend coefficient for each of the later component (i.e., the difference between the first and last ratings). For N2, the linear trend coefficients were above zero for both color photographs and line-drawings, *t*(21) = 5.81, *p* < 0.001, *dz* = 1.24, *B*_N(0,2)_ = 3.84 × 10^5^, *t*(21) = 3.16, *p* < 0.01, *dz* = 0.67, *B*_N(0,2)_ = 27.03. For VAN, the linear trend coefficients were below zero for both types of images, *t*(21) = -4.16, *p* < 0.001, *dz* = 0.89, *B*_N(0,2)_ = 1.13 × 10^3^, *t*(21) = -3.70, *p* = 0.001, *dz* = 0.79, *B*_N(0,2)_ = 3.815 × 10^2^. For LP, there was not much evidence either way for whether or not the linear trend coefficients were different from zero for both types of images, *t*(21) = 1.64, *p* = 0.12, *dz* = 0.35, *B*_N(0,2)_ = 2.56, *t*(21) = 1.31, *p* = 0.20, *dz* = 0.28, *B*_N(0,2)_ = 1.58. Moreover, for N2, “no experience” differed from “brief glimpse” for both color photographs, *t*(21) = -2.80, *p* < 0.05, *dz* = 0.60, *B*_N(0,4)_ = 15.88, and line-drawings, *t*(21) = -2.13, *p* < 0.05, *dz* = 0.45, *B*_N(0,4)_ = 5.14. For VAN, “no experience” also differed from “brief glimpse” for both color photographs, *t*(21) = 3.90, *p* = 0.001, *dz* = 0.83, *B*_N(0,3)_ = 5.84 × 10^2^, and line-drawings: *t*(21) = 2.43, *p* < 0.05, *dz* = 0.52, *B*_N(0,3)_ = 6.08. But for LP, there was marginal evidence for no difference between “no experience” and “brief glimpse” for color photographs, *t*(21) = 0.80, *p* = 0.43, *dz* = 0.17, *B*_N(0,3)_ = 0.37, and evidence for no difference between “no experience” and “brief glimpse” for line-drawings, *t*(21) = 0.26, *p* = 0.80, *dz* = 0.06, *B*_N(0,3)_ = 0.22. The results suggested that the N2 and VAN could be related to PAS in a linear way, whereas the LP correlated with PAS in a non-linear fashion.

**FIGURE 5:**
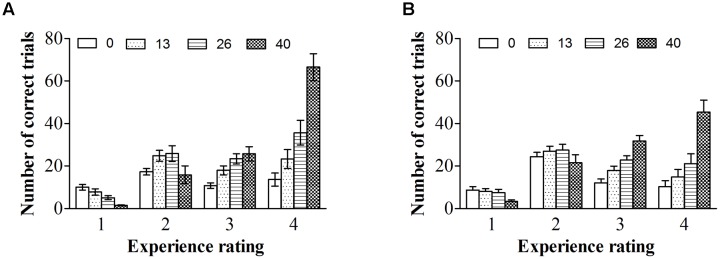
**Interstimulus interval distributions across correct trials for each experience ratings**. **(A)** ISI distributions across correct trials for each experience ratings for color photographs. **(B)** ISI distributions across correct trials for each experience ratings for line-drawings. Error bars depict standard errors.

**FIGURE 6:**
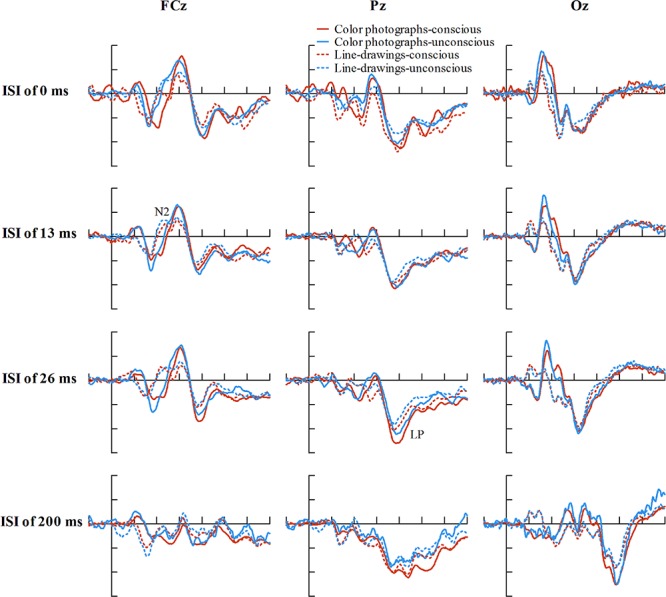
**Grand-average ERPs of correct trials at electrodes FCz, Pz, and Oz for conscious and unconscious category of each ISI for color photographs and line-drawings**. The average number of trials for each condition was 38.01 for color photographs, and 35.43 for line-drawings. (For color photographs, the number of trials for conscious and unconscious category of each ISI was 27.16, 20.26, 32.37, 36.95, 28.26, 54.53, 12.42, 92.16, 33.05, 17.63, 34.05, 28.79, 33.16, 39.68, 21.63, and 75.42, respectively).

To explore the relationship between experience ratings and ERPs controlling for ISIs, we firstly described the distribution of ISIs cross correct trials for each experience rating for color photographs and line-drawings (see **Figure 5**). As can be seen from **Figure 5**, the number of correct trials for some ISI and experience rating combinations was rather small. As ERP effects usually require a large number of trials to measure them accurately in each condition (Luck, 2005, p. 23), “no experience” and “brief glimpse” were collapsed to form an “unconscious” category, while “almost clear experience” and “clear experience” were collapsed together for a “conscious” category, as per Melloni et al. (2011). The data from three participants were excluded because the number of trials for at least one condition was zero for them. **Figure 6** shows the ERP data for conscious and unconscious categories of each ISI for color photographs and line-drawings. An ANOVA with type of image (color photographs vs. line-drawings), experience ratings (unconscious vs. conscious), and ISI (0, 13, 26, and 200 ms) was conducted separately for amplitudes of N2, VAN, and LP. For N2, there was an experience rating effect, *F*(1,18) = 28.91, *p* < 0.001, $\eta_{p}^{2}$ = 0.62, *B*_N(0,4)_ = 2.15 × 10^5^, a significant ISI effect, *F*(3,54) = 20.39, *p* < 0.001, $\eta_{p}^{2}$ = 0.53. For VAN, there was a type of image effect, *F*(1,18) = 26.89, *p* < 0.001, $\eta_{p}^{2}$ = 0.60, *B*_N(0,4)_ = 8.68 × 10^4^, a significant ISI effect, *F*(1.65,29.63) = 37.53, *p* < 0.001, $\eta_{p}^{2}$ = 0.68, and a significant type of image by ISI interaction, *F*(2.34,42.20) = 8.71, *p* < 0.001, $\eta_{p}^{2}$ = 0.33. For LP, there was a type of image effect, *F*(1,18) = 7.50, *p* < 0.05, $\eta_{p}^{2}$ = 0.29, *B*_N(0,3)_ = 8.30, an experience rating effect, *F*(1,18) = 17.67, *p* = 0.001, $\eta_{p}^{2}$ = 0.50, *B*_N(0,3)_ = 9.03 × 10^2^, and a significant ISI effect, *F*(2.21,39.69) = 26.12, *p* < 0.001, $\eta_{p}^{2}$ = 0.59. The main effect of experience ratings on amplitudes indicated that the N2 and LP were each related to subjective awareness even controlling for ISI.

## Discussion

Our behavioral results showed that people reported having more “clear experience” but less “brief glimpse” for color photographs than for line-drawings, suggesting that people had more experienced clarity for color photographs than for line-drawings. Importantly, there was no difference for accuracy between color photographs and line-drawings for each experience rating and the accuracy for both types of image gradually increased with rating, consistent with the experience ratings reflecting visibility. However, the accuracy for both types of images was above chance for “no experience,” providing evidence for subliminal perception with an elaborated subjective scale. This may seem inconsistent with the finding of Ramsøy and Overgaard (2004), in that they found the accuracy for the stimulus form was not significantly better than chance for “no experience”. However, a non-significant result is not in itself evidence for the null hypothesis.

The ERP results showed that the posterior P1 amplitude did not change with the PAS ratings, confirming previous findings that the early P1 component is not related to visual awareness (Del Cul et al., 2007; Koivisto et al., 2008; Melloni et al., 2011). Further, the posterior N1 and anterior P2 components did not vary with the PAS ratings for line-drawings at least. The N1 amplitude for color photographs decreased with increasing subjective ratings, consistent with previous results showing that the N1 amplitude decreased when visibility increased (Melloni et al., 2011). However, as the N1 component for line-drawings did not vary with subjective ratings, the N1 component cannot be a general indicator of subjective awareness. Similarly, the P2 amplitude for color photographs decreased with increasing subjective ratings, consistent with previous findings that the P2 amplitude decreased as visibility increased (Melloni et al., 2011). Nonetheless, as the P2 component for line-drawings did not change with subjective ratings, indicating that the P2 component is also not always related to subjective awareness. It has been found that N1 and P2 are associated with feature detection or integration (Hillyard and Münte, 1984; Luck and Hillyard, 1994). The features were more complex in color photographs than in line-drawings. Thus, the fact that N1 and P2 correlated with experience ratings for color photographs rather than for line-drawings may be because that the feature analysis varied with the experience ratings for color photographs but not for line-drawings.

Importantly, we found that the posterior VAN did vary with the PAS ratings linearly for both color photographs and line-drawings, which is consistent with previous findings that the posterior VAN correlated with visual awareness, as the recurrent process hypothesis theory proposes (Koivisto and Revonsuo, 2003; Koivisto et al., 2008; Genetti et al., 2009). Indeed, Koivisto and Revonsuo (2010) reviewed ERP studies on visual awareness and suggested that VAN is the most consistently observed feature across different studies. However, we found that there were significant differences for the VAN amplitudes between “no experience” and “brief glimpse” and between “brief glimpse” and “almost clear experience” for both color photographs and line-drawings. On the view that “no experience” and “brief glimpse” can be collapsed together as reflecting lack of conscious awareness of visual features relevant to the task discrimination (Dienes and Seth, 2010), the VAN amplitude increase would not be a good indicator of the emergence of subjective awareness.

Moreover, our results showed that the anterior N2 gradually decreased with the PAS ratings. The anterior N2 has been observed to correlate with conscious awareness in other cognitive tasks. For example, Eimer et al. (1996) found that the anterior N2 enhancement for deviant stimuli compared with standard stimuli might be regarded as an indicator of the amount of explicit (conscious) knowledge in a serial reaction task. Schankin and Wascher (2007) also found that the anterior N2 were smaller for detected or aware changes than for undetected or unaware changes in Experiment 1 in a change detection task. Nonetheless, Schankin and Wascher (2007) did not observe the N2 effect when the influence of working memory was eliminated in Experiment 2, and a few studies have found that the N2 amplitudes were not significantly affected by subjectively awareness of the targets when they were simple lines or shapes (e.g., Koivisto and Revonsuo, 2003; Lamy et al., 2009). As N2 is related to an actively attended mismatch between a stimulus and a mental template (Folstein and Van Petten, 2008), the inconsistent findings may be due to the requirement difference on memory retrieval in different tasks.

Unlike VAN and N2 amplitudes, the P3a and LP (or P3) amplitudes seemed to change non-linearly with subjective ratings. For color photographs, the P3a amplitude was not larger for “brief glimpse” than for “almost clear experience,” suggesting that the P3a was not sensitive to subjective awareness. As P3a reflects top-down monitoring by frontal attention mechanisms engaged to evaluate incoming stimuli (Polich, 2007), the P3a amplitude might be related to evaluating difficulty more than subjective awareness. Importantly, the LP amplitude was larger for “almost clear experience” than for “brief glimpse,” but there was no difference between “brief glimpse” and “no experience,” indicating that the LP amplitude is an indicator of the emergence of subjective awareness, assuming a non-linear relation between PAS and relevant awareness. The results indicated that visual awareness is correlated with later activation indicated by P3 or LP, as suggested by the GWS and HOT theories (Koivisto et al., 2008; Genetti et al., 2009).

The different patterns of VAN and LP in our findings are consistent with different stages or types of subjective awareness. It has been noted that the perception of masked displays might involve two stages: during the early stage, i.e., the first 200 to 300 ms from the stimulus onset, the brain responses detectable with ERPs increase linearly with the stimulus energy or duration, whereas in the second stage, after approximately 300 ms from the stimulus onset, the brain activity detectable with ERPs is characterized by a non-linear relation to stimulus magnitudes (see Kouider et al., 2013). Koivisto and Revonsuo (2003) suggested that the VAN is an electrophysiological correlate of phenomenal visual awareness, the subjective experience of seeing the stimulus, whereas the later LP reflects access visual awareness, the conscious evaluation of the stimulus for decision-making (Block, 1995, 1996). Lamme (2010) also argued that all the neural ingredients that seem to matter for the visual phenomenality are present in the localized recurrent processes (indicated by the VAN), and the widespread recurrent processes (indicated by the LP) allow to have only reportable conscious visual information. Therefore, the VAN may change linearly with the quality of visual perception, which may reflect how first order visual quality, which some may call visual phenomenal awareness, is graded or continuous; whereas the LP varies non-linearly with PAS, and may reflect awareness of being in a relevant mental state (and therefore the state being conscious by higher order and GWS theories).

Finally, we should note some limitations in the present study. First, as different experience ratings included different proportion of trials with same ISIs, there is an issue of the influence of ISI on visibility. Ideally, the physical stimulation should remain constant when compared trials with different experience ratings. However, as a sufficient amount of responses for each experience rating was necessary, different contrasts between the target and the background (e.g., Andersen et al., 2015) or different stimulus durations (e.g., Koivisto and Grassini, 2016) were often used. In the present study, the stimulus duration was same for all experience ratings, while the ISI between the target and the mask was varied. Previous studies revealed that backward masking does not influence the feed-forward processing but interrupts the recurrent processing between high and low cortical areas which is crucial for the emergence of visual awareness (Lamme et al., 2002). Importantly, when “no experience” and “brief glimpse” were collapsed to form an “unconscious” category and “almost clear experience” and “clear experience” to form a “conscious” category, we also found that N2 and LP were each related to subjective awareness even controlling for ISI. However, further studies could still usefully explore how VAN and LP vary with experience ratings when the ISI remains unchanged so the control is not just statistical. Second, although we attempted to find neural correlates of subjective awareness, we could not assume that participants always provided accurate descriptions of their experience in their subjective reports (Dehaene and Naccache, 2001). Fortunately, we found that there were no significant accuracy differences between color photographs and line-drawings for each experience rating, suggesting that the rating was relatively accurate in the present study. Our findings are the first to show that the VAN amplitude increased linearly with the PAS ratings whereas the LP amplitude increased non-linearly with PAS ratings, which is helpful to reconcile the apparently contradictory theories and to resolve the current debate on the neural correlates of visual awareness.

## Ethics Statement

This experiment was approved by the committee for the protection of subjects at the Institute of Psychology, Chinese Academy of Sciences. All students gave written informed consent and were paid for their attendance.

## Author Contributions

QF, Y-JL, WC, and XF designed the experiment. QF, Y-JL, and WC prepared materials and performed the experiment. QF, ZD, and JW analyzed the data, and QF, ZD, JW, and XF wrote the paper.

## Conflict of Interest Statement

The authors declare that the research was conducted in the absence of any commercial or financial relationships that could be construed as a potential conflict of interest.
